# Sequence-Based SSR Marker Development and Their Application in Defining the Introgressions of LA0716 (*Solanum pennellii*) in the Background of cv. M82 (*Solanum lycopersicum*)

**DOI:** 10.1371/journal.pone.0081091

**Published:** 2013-12-05

**Authors:** Wenbo Long, Ye Li, Wenjuan Zhou, Hong-Qing Ling, Shusong Zheng

**Affiliations:** State Key Laboratory of Plant Cell and Chromosome Engineering, Institute of Genetics and Developmental Biology, Chinese Academy of Sciences, Beijing, China; Nanjing Agricultural University, China

## Abstract

The introgression lines (ILs) from cv. M82 (*Solanum lycopersicum*) × LA0716 (*S. pennellii*) have been proven to be exceptionally useful for genetic analysis and gene cloning. The introgressions were originally defined by RFLP markers at their development. The objectives of this study are to develop polymorphic SSR markers, and to re-define the DNA introgression from LA0716 in the ILs. Tomato sequence data was scanned by software to generate SSR markers. In total, 829 SSRs, which could be robustly amplified by PCR, were developed. Among them, 658 SSRs were dinucleotide repeats, 162 were trinucleotide repeats, and nine were tetranucleotide repeats. The 829 SSRs together with 96 published RFLPs were integrated into the physical linkage map of *S. lycopersicum*. Introgressions of DNA fragments from LA0716 were re-defined among the 75 ILs using the newly developed SSRs. A specific introgression of DNA fragment from LA0716 was identified in 72 ILs as described previously by RFLP, whereas the specific DNA introgression described previously were not detected in the ILs LA4035, LA4059 and LA4091. The physical location of each investigated DNA introgression was finely determined by SSR mapping. Among the 72 ILs, eight ILs showed a shorter and three ILs (IL3-2, IL12-3 and IL12-3-1) revealed a longer DNA introgression than that framed by RFLPs. Furthermore, 54 previously undefined segments were found in 21 ILs, ranging from 1 to 11 DNA introgressions per IL. Generally, the newly developed SSRs provide additional markers for genetic studies of tomatoes, and the fine definition of DNA introgressions from LA0716 would facilitate the use of the ILs for genetic analysis and gene cloning.

## Introduction

The tomato (*Solanum lycopersicum* L.) has a rich genetic diversity in terms of cultivars and especially its wild relatives. Wild germplasms, such as *S. pennellii* [1], *S. pimpinellifolium* [2], and *S. habrochaites* [3], [4] have been used in breeding and genetic studies. *S. pennellii*, famous for its high drought tolerance, self-compatibility and easy hybridization to *S. lycopersicum* [5], was used in many studies, such as gene mapping and QTL analysis [6], the research on the mechanism of reproductive barriers in plant [7], [8], abiotic stresses especially salt tolerance [9], [10]. A population of 76 introgression lines (ILs, BC_4_ using M82 as the recurrent parent) were developed from a cross between *S. pennellii* (LA0716) × *S. lycopersicum* (cv. M82) and were selected using restriction fragment length polymorphism (RFLP) markers [1], [6]. The 76 ILs contained overlapping chromosome segments that covered the donor genome of LA0716, and each IL was almost isogenic to the domesticated tomato (M82), except for the *S. pennellii* introgression. All introgression segments were defined by RFLP markers [6]. Recently, Van Schalkwyk *et al*. [11] mapped 66 ILs in the population with 990 polymorphic diversity array technology (DArT) markers and 108 RFLP markers. Each LA0716 introgression was mapped and placed into bins using an average of 10 markers. Sim *et al.* [12] located most introgressions from LA0716 (except parts of chromosomes 4, 5, 8 and 9) in 76 ILs with 3504 nucleotide polymorphism markers (SNPs). The 76 ILs have been widely employed in genetic studies for research in many areas, including the QTL analysis of fruit and yield related traits [13], [14], cloning and heterosis analysis of the self-pruning gene [15], [16], fine mapping of the carotenoid-related candidate gene [17], metabolic mechanisms [18], and ascorbic acid-related genes [19].

Diverse molecular markers have been developed for tomato research, including RFLP [5], [6], the retrotransposon-based sequence-specific amplification polymorphism (SSAP) [20], random amplified polymorphic DNA (RAPD) [21], [22], amplified fragment length polymorphism (AFLP) [20], simple sequence repeat (SSR) [23], [24], [25], [26], DArT [11] and SNP [12], [27]. Among these, RFLP markers were commonly used to construct linkage maps of tomato in the 1990s [13], [28]. However, RFLP analysis is rather laborious and requires autoradiography equipment. Recently, with the fast development of next generation sequencers, huge numbers of SNPs were detected among tomato wild relatives, germplasms and varieties [12], [27]. High throughput SNP genotyping arrays were developed for scoring single SNP in thousands of different samples or millions of SNPs in a single sample [27]. However, at this time, this technology remains for laboratories with special equipments and good finical support. In contrast, the SSR marker (microsatellite) has high polymorphism and is simple to use, and has therefore been used widely in genetic analysis of many crop species. For the tomato, the first set of 150 SSR markers were identified by screening tomato EST sequences against *Arabidopsis*, which were then mapped to the Tomato-EXPEN 2000 mapping population, derived from the cross between LA0925 (*S. Lycopersicum*) × LA0716 (*S. pennellii*) [29]. Ohyama *et al*. [26] generated 148 SSRs from 89,824 cDNA sequences and 537 SSRs from 310,583 BAC-ended sequences, and also mapped these SSRs to the Tomato-EXPEN 2000 population. Shirasawa *et al*. [30] developed 2047 SSRs from tomato expressed sequence tags and 3510 SSRs from tomato genome sequences. Among the 5557 SSRs, 1433 were mapped to the Tomato-EXPEN 2000 population. Additionally, 54 and 37 SSRs were developed from tomato-anchored BAC-clones of chromosomes 6 and 12, respectively [23], [24]. Thus, there are almost 2400 mapped SSRs available for tomato genetic analysis at present. In this study, we developed polymorphic SSRs between LA0716 and M82 from tomato genome sequence [31], and defined introgressions from LA0716 in the 76 ILs of the LA0716 × M82 population using these SSRs.

## Materials and Methods

### Plant Materials

Seventy five ILs (no seed for LA4061) derived from the cross between LA0716 × M82 [6], and their parents cv. M82 (LA3475, *S. lycopersicum*) and LA0716 (*S. pennellii*) were used in this study. All seeds were from the Tomato Genetics Resource Center (TGRC) at the University of California (Davis, United States of America) in 2003. In 2010, 14 ILs which either failed to exhibit the target introgressions from LA0716 or which had undefined segment(s) were reordered from TGRC to confirm our results; these included accessions LA3480 (LA4035), LA3501 (LA4060), LA4031, LA4037, LA4038, LA4044, LA4055, LA4059, LA4060, LA4062, LA4063, LA4090, LA4091, and LA4094. Tomato plants were grown in the greenhouse and experimental field of Institute of Genetics and Developmental Biology, Chinese Academy of Science. The genomic DNA of each accession was isolated from young leaves of a single plant following the modified CTAB method described by Stewart and Via [32]. At least five plants for each IL were analyzed for determination of the DNA introgression.

### Development of Polymorphic SSRs from Tomato Released Sequences

Tomato sequence data was downloaded from the Sol Genomics Network (http://solgenomics.net). Simple repeated sequences in tomato scaffolds were identified using the SSRhunter 1.3 software [33]. Primer pairs for PCR amplification were designed using Primer Premier 5.0 [34]. A few SSR loci were adopted from the Tomato-EXPEN 2000 linkage map [29] and the linkage map developed by Shirasawa *et al*. [30]. PCR was performed in a total volume of 25 µl containing 40–60 ng DNA template, 1 × PCR buffer (20 mM Tris pH 9.0, 100 mM KCl, 2.0 mM MgCl_2_), 200 µM of each of the four dNTPs, 0.2 µM of each of the forward and reverse primers, and one unit of rTaq DNA polymerase (Takara Bio-incorporation). The PCR reactions were carried out as follows: an initial 5 min at 94°C, 35 cycles of 94°C for 30 s, 45–55°C for 15–30 s, 72°C for 20 s and a final 5 min at 72°C. PCR products were separated on 9% polyacrylamide gels using a smart electrophoresis system (DYCZ-30B electrophoresis cell) at 300 V and screened by the silver-stain method. Firstly, all SSRs were tested for their polymorphism on M82 and LA0716, and the polymorphic SSRs were then used to detect the DNA introgressions of LA0716 in the IL population of LA0716 × M82.

### Physical Distance Map Construction

Physical distances between SSRs were calculated based on the tomato sequence (http://solgenomics.net). Physical linkage maps of all 12 linkage groups were constructed using the newly developed 829 SSRs together with the 96 published RFLPs used as anchors [1], [6]. Maps were constructed using the Mapdraw 3.0 software [35]. The euchromatic and heterochromatic regions in each linkage group were roughly estimated according to the position of markers anchored to the heterochromatin reported previously [30], [36], [37].

### Identification of Introgressions

The location of each introgression in the 75 ILs of LA0716 × M82 was roughly estimated according to previous RFLP results [6]. To find the two ends of each specific introgression, PCR amplification of consecutive SSRs along the introgression segment was performed on the DNA templates of the corresponding IL and two parents (M82 and LA0716) until the last two SSRs amplifying LA0716-specific PCR products were identified. The physical size of each introgression was determined by the physical distance between the two SSRs located at the ends of the segment. The IL maps were constructed using the Mapdraw 3.0 software [35]. To detect IL segments that were not defined by the RFLPs [6], 179 SSRs, which were evenly distributed on the 12 chromosomes with a 5–10 cM distance between two adjacent SSRs, were amplified by PCR using the 75 ILs and the parents (M82 and LA0716) as DNA templates.

### Analysis of the Relative Genetic Size and Relative Physical Size of Introgressions

To compare the genetic and physical sizes of the introgressions and to investigate the segregation distortion of introgressions from the LA0716 × M82 cross, the relative genetic and physical sizes were calculated as follows: relative genetic size (RGS) = introgression genetic size/chromosome total genetic size (excluding the heterochromatic region); relative physical size (RPS) = introgression physical size/chromosome total physical size (excluding the heterochromatic region).

## Results

### Development and Characteristics of the SSR Markers

In total, 6000 SSR sites across the tomato genome were investigated, 1670 of them produced a clear banding pattern of DNA fragments with the expected sizes following PCR analysis, and 1328 SSRs showed polymorphism between M82 and LA0716. Of the polymorphic SSRs, 829 (62.4%) were highly reproducible with a size ranging from 70 to 250 bp (an easily separated size in a polyacrylamide gel) (Table S1) and were used for the further characterization.

Among the 829 SSRs, 658 (79.4%) SSRs were dinucleotide repeats, 162 (19.5%) were trinucleotide repeats, and nine (1.1%) were tetranucleotide repeats (Table 1). The poly (AT)n motif comprised the majority of the dinucleotide repeats (552 SSRs, 83.9%), followed by poly (AG)n (71 SSRs, 10.8%), poly (AC)n (33 SSRs, 5.0%) and poly (GC)n (2 SSRs, 0.3%). We also found more than eight types of trinucleotide repeat; the AT-rich motifs, namely poly (ATT)n, (ATA)n and (ATC)n, were detected more frequently than other motifs, and comprised 74.7% of the trinucleotide repeats. The PCR product sizes of the 678 SSR alleles (81.8%) were 100–200 bp, with only 71 SSRs (8.6%) being over 200 bp, and 80 SSRs (9.7%) less than 100 bp. Among the 829 SSRs, 466 (56.2%) showed co-dominant polymorphisms. LA0716 had a total of 89 SSRs (10.7%) with null alleles, whereas 274 SSRs (33.1%) with null alleles were observed in M82 (Table S1).

**Table 1:** Microsatellite repeat motifs of the 829 SSR markers.

SSR Motif	Chr1	Chr2	Chr3	Chr4	Chr5	Chr6	Chr7	Chr8	Chr9	Chr10	Chr11	Chr12	Total
Di-nucleotides													
AT	55 (64.7^a^)	67 (61.5)	43 (60.6)	47 (62.7)	47 (77.0)	36 (78.3)	42 (72.4)	44 (72.1)	53 (60.9)	40 (71.4)	31 (53.4)	47 (74.6)	552 (66.5)
AG	11 (12.9)	8 (7.3)	7 (9.9)	12 (16.0)	2 (3.3)	4 (8.7)	5 (8.6)	5 (8.2)	6 (6.9)	2 (3.6)	6 (10.3)	4 (6.3)	71 (8.6)
AC	2 (2.4)	3 (2.8)	7 (9.9)	3 (4.0)	1 (1.6)	1 (2.2)	2 (3.4)	2 (3.3)	4 (4.6)	0 (0.0)	7 (12.1)	1 (1.6)	33 (4.0)
CG	0 (0.0)	1 (0.9)	0 (0.0)	0 (0.0)	0 (0.0)	0 (0.0)	0 (0.0)	1 (1.6)	0 (0.0)	0 (0.0)	0 (0.0)	0 (0.0)	2 (0.2)
Subtotal	68 (80.0)	78 (71.6)	57 (80.3)	62 (82.7)	50 (82.0)	41 (89.1)	49 (84.5)	52 (85.2)	63 (72.4)	42 (76.4)	44 (75.9)	52 (82.5)	658 (79.4)
Tri-nucleotides													
ATT	6 (7.1)	11 (10.0)	5 (7.0)	8 (10.7)	3 (4.9)	2 (4.3)	5 (8.6)	4 (6.6)	17 (19.5)	6 (10.7)	8 (13.8)	4 (6.3)	79 (9.5)
ATA	5 (5.9)	6 (5.5)	1 (1.4)	4 (5.3)	3 (4.9)	1 (2.2)	1 (1.7)	3 (4.9)	3 (3.4)	4 (7.1)	3 (5.2)	7 (11.1)	41 (4.9)
AAG	4 (4.7)	7 (6.4)	0 (0.0)	0 (0.0)	1 (1.6)	0 (0.0)	1 (1.7)	2 (3.3)	2 (2.3)	0 (0.0)	0 (0.0)	0 (0.0)	18 (2.2)
AAC	2 (2.4)	1 (0.9)	1 (1.4)	0 (0.0)	0 (0.0)	0 (0.0)	1 (1.7)	0 (0.0)	1 (1.1)	0 (0.0)	2 (3.4)	0 (0.0)	8 (1.0)
ATC	0 (0.0)	0 (0.0)	0 (0.0)	0 (0.0)	0 (0.0)	0 (0.0)	1 (1.7)	0 (0.0)	0 (0.0)	0 (0.0)	0 (0.0)	0 (0.0)	1 (0.1)
AGA	0 (0.0)	0 (0.0)	1 (1.4)	0 (0.0)	1 (1.7)	0 (0.0)	0 (0.0)	0 (0.0)	0 (0.0)	1 (1.8)	0 (0.0)	0 (0.0)	3 (0.4)
ACA	0 (0.0)	1 (0.9)	0 (0.0)	0 (0.0)	1 (1.7)	0 (0.0)	0 (0.0)	0 (0.0)	0 (0.0)	0 (0.0)	0 (0.0)	0 (0.0)	2 (0.2)
CGG	0 (0.0)	1 (0.9)	0 (0.0)	0 (0.0)	0 (0.0)	1 (2.2)	0 (0.0)	0 (0.0)	0 (0.0)	1 (1.8)	0 (0.0)	0 (0.0)	3 (0.4)
others	0 (0.0)	1 (0.9)	3 (4.2)	1 (1.3)	0 (0.0)	1 (2.2)	0 (0.0)	0 (0.0)	0 (0.0)	0 (0.0)	1 (1.7)	0 (0.0)	7 (0.8)
Subtotal	17 (20.0)	29 (26.6)	11 (15.5)	13 (17.3)	9 (14.8)	5 (10.9)	9 (15.5)	9 (14.8)	23 (26.4)	12 (21.8)	14 (24.1)	11 (17.5)	162 (19.5)
Tetranucleotide	0 (0.0)	2 (1.8)	3 (4.2)	0 (0.0)	2 (3.3)	0 (0.0)	0 (0.0)	0 (0.0)	1 (1.1)	1 (1.8)	0 (0.0)	0 (0.0)	9 (1.1)
Total	85 (100)	109 (100)	71 (100)	75 (100)	61 (100)	46 (100)	58 (100)	61 (100)	87 (100)	55 (100)	58 (100)	63 (100)	829 (100)

a Numbers in parentheses indicate the percentage of the total.

### Construction of Physical Distance and ILs Maps

**Figure 1:**
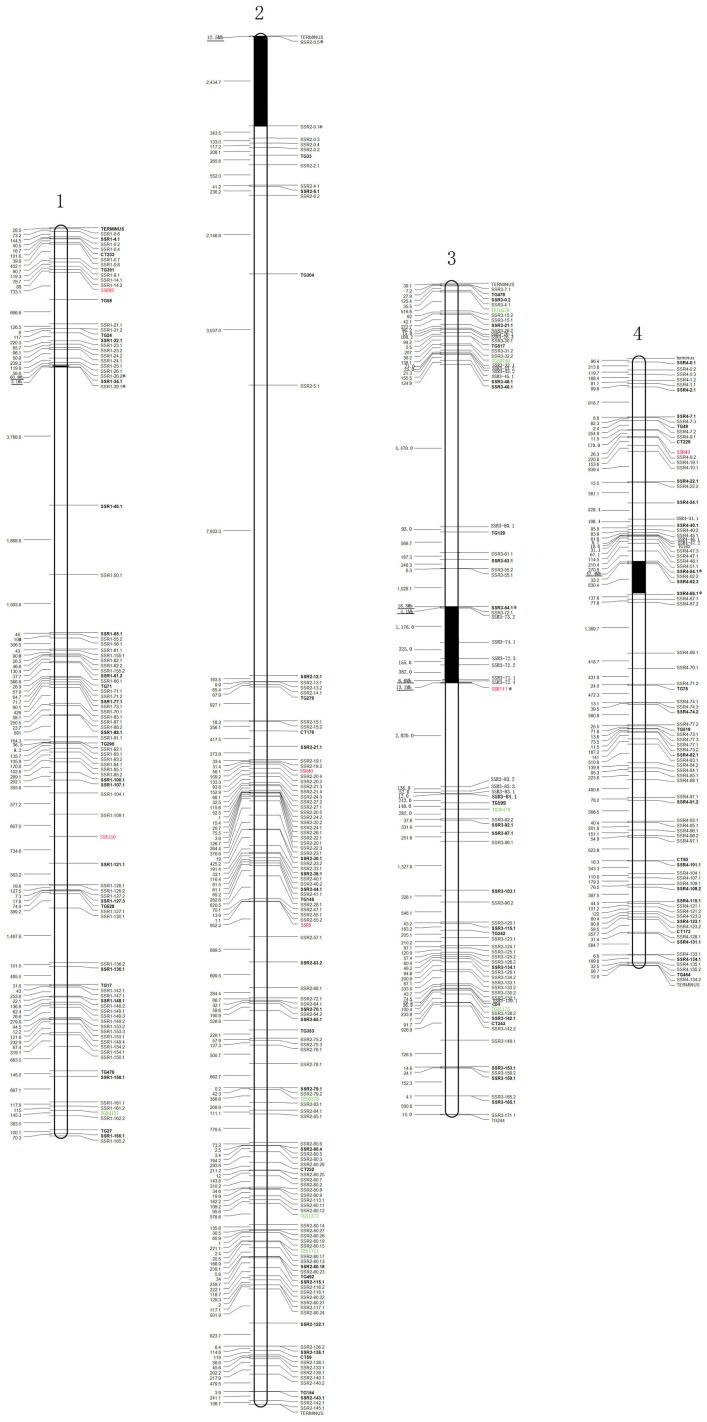
Physical linkage map of the tomato genome. Twelve linkage groups were constructed using 829 SSR markers (SSRs) and 96 RFLP markers (RFLPs). The names of the SSRs are marked to the right of each chromosome and the inter-distance in kb between each two adjacent SSRs is marked on the left. The Inter-distance in the figure means the physical distance between each two adjacent SSR markers and the black bar under inter-distances highlights some very large inter-distances with measurement unit Mb instead of kb. The markers in the bold font are the 96 RFLPs from the Tomato IL Map on the Sol Genomics Network (http://sgn.cornell.edu/), and the 179 SSRs investigated previously undefined segments in 75 introgression lines from the cross between cv. M82 (*S. lycopersicum*) × LA0716 (*S. pennellii*). The thick black bar indicates the presumed heterochomatin region, between two SSRs marked with *. Euchromatin and heterochromatin regions were estimated based on previous reports and the released sequence data of the tomato genome on the Sol Genomics Network (http://sgn.cornell.edu/). The SSR loci adopted from Tomato-EXPEN2000 Map [29] and the linkage map constructed by Shirasawa et al. [30] were marked with red and green color, respectively.

All 829 SSRs were mapped to 12 linkage groups with 96 published RFLP markers corresponding to the 12 chromosomes (Figure 1). The 829 SSRs and 96 RFLPs were integrated into a physical distance map with an average of 69 SSRs per linkage group, ranging from 46 to 109 SSRs in one linkage group. Among the 829 SSRs, 789 SSRs (95.2%) were found in euchromatic regions and only 40 (4.8%) in heterochromatic regions. The distance between two SSRs was calculated based on the tomato DNA sequence. In total, the inter-distances among the 829 SSRs added up to 744.4 Mb, including 242.8 Mb in the euchromatin regions and 501.6 Mb in the heterochromatin regions. The average distance between every two SSRs was 898.0 kb including both heterochromatin and euchromatic regions, whereas the average distance was reduced to 291.6 kb when calculated without heterochromatin regions.

**Figure 2:**
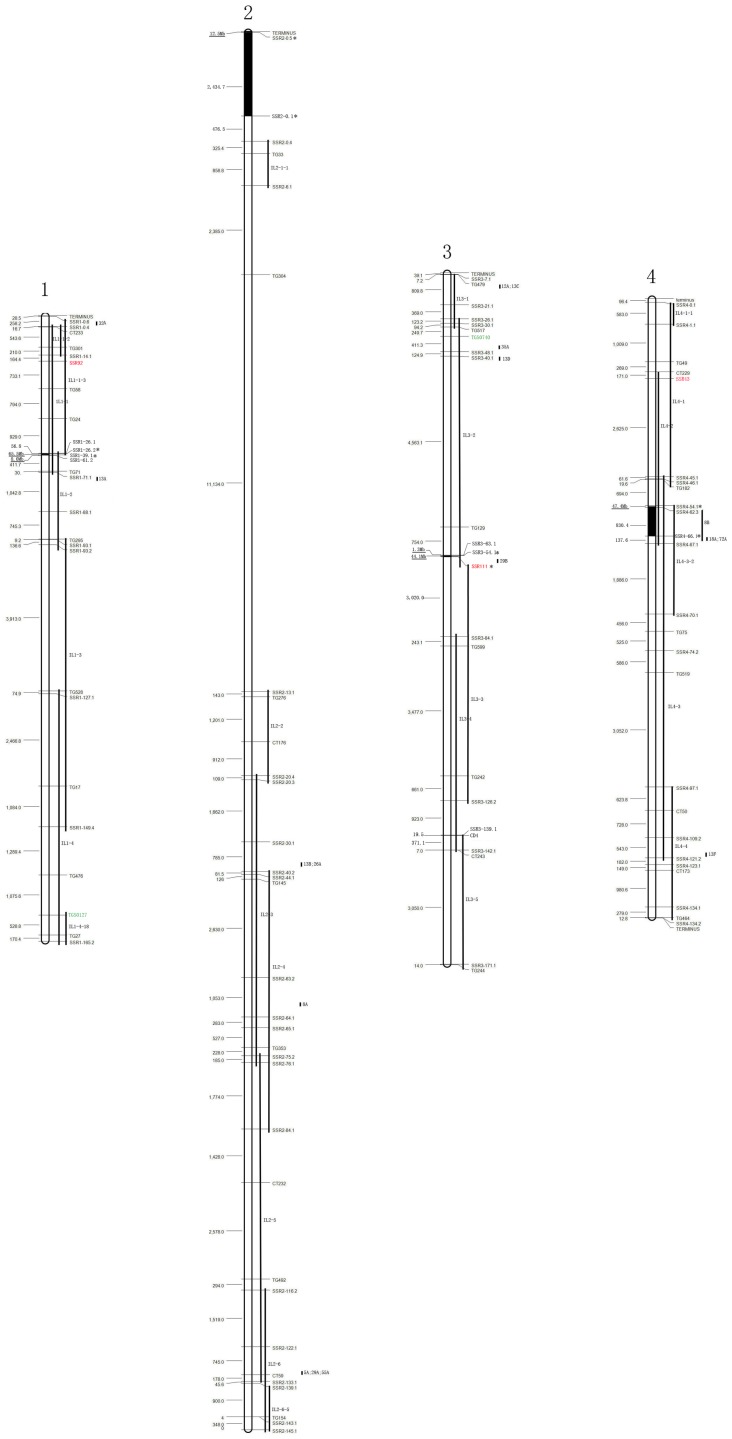
Linkage map of introgressions in 75 introgression lines (ILs) from the cross between cv. M82 (*S. lycopersicum*) × LA0716 (*S. pennellii*). Twelve linkage groups were constructed using 96 RFLP markers (RFLPs) from the Tomato IL Map on the Sol Genomics Network (http://sgn.cornell.edu/) and 168 SSR markers (SSRs) used to define the ends of each introgression and previously undefined loci in 75 introgression lines (ILs). The bars in the right side indicate the introgression segments in the ILs. The furthest short bars in the right side of each chromosome indicate the previously undefined DNA segments. The inter-distance in kb between each two adjacent SSRs is marked on the left. The black bar under the inter-distances highlights some very large inter-distances with measurement unit Mb instead of kb. The thick black bar indicates presumed heterochromatin region between two SSRs marked with *. Euchromatin and heterochromatin regions were estimated based on previous reports and the released sequence data of the tomato genome on the Sol Genomics Network (http://sgn.cornell.edu/). The SSR loci marked with red color were adopted from Tomato-EXPEN2000 Map [29], and the SSRs with green color were shared by the linkage map developed by Shirasawa et al. [30].

Introgressions from LA0716, which were previously defined by RFLPs [6], were scanned by SSRs in this study. All 75 ILs, except for LA4035, LA4059, and LA4091, were found with introgressions from LA0716 as defined previously by RFLPs. Most of the physical locations of the introgressions from LA0716 found using SSRs were approximately equivalent to the bins framed by the RFLPs (Figure 2, Table S2). However, eight introgressions from LA0716 were found to be shorter than the bins framed by the RFLPs, and these included IL4-3-2, IL5-2, IL6-3, IL7-4, IL8-1, IL9-2-6, IL10-2, and IL12-4. Another three introgressions (IL3-2, IL12-3, and IL12-3-1) were found to be longer than the bins delimited by the RFLPs. We failed to identify IL2-1 in LA4035, IL6-1 in LA4059, and IL10-3 in LA4091, even after analyzing at least six individuals for each of the 3 ILs.

The physical size of each introgression was calculated by the distance between two delimited SSRs for each segment (Table S2). Among the 72 detected introgressions, 45 ranged in size between 1–10 Mb; ten introgressions were less than 1 Mb, including IL1-1-2, IL1-4-18, IL4-1-1, IL6-4, IL8-3-1, IL9-2-5, IL9-2-6, IL11-4-1 IL12-1-1, and IL12-4-1; 17 introgressions, which contained a centromere region, were extremely large, ranging from 44.4–77.4 Mb. The largest introgression IL1-1 (77.4 Mb) was located in linkage group 1, while the smallest IL12-4-1 (300 kb) was in linkage group 12.

In total, 69 introgressions were homozygous in all detected individuals, but three introgressions (IL2-3, IL2-4, and IL9-3) were heterozygous in some individuals. Centromere regions were covered by one or more introgressions, except in chromosomes 2 and 6. Eight chromosomes were almost completely covered by overlapping introgressions. However, the coverage of chromosomes 2, 6, 8, and 10 was 41.9%, 20.9%, 97.2% and 96.0%, respectively. The low coverage of chromosomes 2 and 6 was mainly due to the absence of IL2-1 and IL6-1.

### Investigation of Undefined DNA Introgressions in ILs

Undefined introgressions of DNA segments were discovered in 21 ILs (21/75, 28%) by 179 SSRs. The total number of previously undefined introgressions was 54, ranging from 1–11 in one IL (Figure 2 and Table S2). We could not detect the introgressions of IL2-1 in LA4035 and of IL6-1 in LA4059, which were defined by RFLPs in a previous study [6]. However, we found undefined DNA segments from LA0716 in the two lines, and in another 19 ILs. Both the specific introgressions defined by RFLPs and the undefined segments were from LA0716. Thirteen ILs (13/21, 61.9%) contained only one undefined DNA segment. Two lines (LA4035 and LA4082) were found to contain two undefined DNA segments, while the line LA4069 contained three undefined DNA segments. Five lines (5/21, 23.8%), including LA4099, LA3494, LA3497, LA4038 and LA4040, contained 4, 6, 6, 7, and 11 undefined DNA segments, respectively. Among the 54 previously undefined DNA segments, 51 (94.4%) were detected by only one SSR marker of the tested 179 SSRs, so their sizes could not be calculated. The other three segments (8B, 11F and 75A) were detected by two or more SSRs (Figure 2), and their sizes were calculated to be 830 kb and 693 kb, and 46.8 Mb, respectively.

## Discussion

In the present study, 829 SSRs were developed using the released tomato sequences, and revealed a polymorphic fragment length between LA0716 and M82. Among the 829 SSRs, 803 (96.9%) were newly mapped onto tomato linkage groups and the other 26 SSRs matched SSR loci of either the Tomato-EXPEN2000 Map on the Sol Genomics Network (http://sgn.cornell.edu/) or the linkage map constructed by Shirasawa *et al*. [30] (http://www.kazusa.or.jp/tomato/). Among the 829 mapped SSRs, 43.8% (363/829) SSRs exhibited dominant polymorphism, which was lower than that (71%) found by Shirasawa *et al*. [30]. In their study, most null alleles were observed in LA0716, whereas in our study, only 24.3% of null alleles (89/363 dominant SSRs) were found in LA0716; the remainder were in M82.

In this study, it was not our intention to select dinucleotide repeat loci for the SSR development; however, about 70% of the SSR motifs were dinucleotides, and 28% were trinucleotides, among the 6000 SSR loci investigated. Comparatively, in the mapped 829 SSRs, 658 SSRs (79.4%) were dinucleotide repeat motifs and only 162 SSRs (19.5%) were trinucleotides (Table S1). Similarly, dinucleotide repeat motifs were predominant in the polymorphic SSRs, followed by trinucleotide repeats, based on the genomic sequences of the anchored tomato BAC clones on chromosomes 6 and 12 [14], [16]. However, among the 13,501 tomato SSRs extracted from 90,763 BAC-end sequences, 7,005 SSRs (51.8%) were trinucleotide repeats, 2,338 (17%) were dinucleotide repeats and 4,158 (31%) were tetranucleotide repeats [30]. Therefore, trinucleotide SSRs accounted for the highest percentage of total SSRs in the tomato at the genome level. This raised the question as to why SSRs containing dinucleotide repeats were predominant in the polymorphic SSRs? We analyzed the simple repeat sequence lengths of the 6000 loci, and found they were mostly greater than 10 bp. It is well known that the length is an important factor in identification of SSRs. Trinucleotide repeat sequences comprised a higher percentage of the total repeat sequences at the genomic level, but a lower percentage of the SSR motifs greater than 10 bp. We examined the 829 SSRs, and found the average number of dinucleotide repeats per motif was 13.4 (26.8 bp in length), while for trinucleotides it was 9.9 (29.7 bp in length). However, for the 6000 SSR loci, the average number of dinucleotide repeats per motif was 9.7 (19.4 bp in length) while that for trinucleotides was only 6.5 (19.5 bp in length). The increased numbers of repeats of both the dinucleotides and trinucleotides in polymorphic SSRs suggest that the higher the number of repeats of the SSR motif, the higher the possibility of variation in the SSRs. We also found a predominance of the poly (AT)n motif in the dinucleotide repeats (552/658 dinucleotide SSRs, 83.9%), and AT/TA sequence in trinucleotide repeats (121/162 trinucleotide SSRs, 74.7%). This indicated that dinucleotide and trinucleotide SSRs containing high repeat numbers, especially those containing the AT/TA motif, might be associated with a higher sequence variation frequency in tomato germplasms. This should be taken into account when developing SSRs.

When Eshed *et al*. [1], [6] established introgression lines from the cross between LA0716 × M82, RFLPs were used as delimiting markers to anchor the bins and analyze the genetic sizes of each introgression in ILs. RFLP was a codominant marker that facilitated the detection of introgressions in ILs. However, RFLP is laborious, and the frame of each introgression could not be intensively scanned because of the limited number of RFLPs and the lack of genomic sequence data at that time. Recently, Van Schalkwyk *et al*. [11] applied DArT markers on 75 ILs, and bin-mapped 66 ILs with 990 polymorphic DArT markers using previously defined RFLP markers as anchors, but failed to found introgressions in the rest nine ILs because of lacking polymorphic DArT markers. Sim *et al.* [12] scanned 76 ILs with 3504 SNPs that were polymorphic between M82 and LA0716 using a SNP array. They successfully located 70 introgressions in the ILs, but did not find rest six segments IL5-2, IL7-5-5, IL8-1-3, IL9-1-3, IL9-2 and IL9-2-5. Among those detected introgressions, large SNP gaps (at least 5 cM) exited in 30 ILs such as IL3-1(19.2 cM gap), IL4-3 (10.2 cM gap), IL9-3 (14.9 cM gap), IL12-3 (13.7 cM gap) and so on.

Though the some introgressions can be traced by CAPs markers converted from DArT markers developed by Van Schalkwyk *et al*. [11], or by SNP markers identified by Sim *et al.* [12], a more straightforward testing method need to be developed for research groups that need to quickly screen research/breeding populations that developed with these ILs.

In this study, we have redefined introgressions using 829 SSRs. As a result, the frames of introgression defined by SSRs in this study are a bit longer than those defined by RFLPs in most cases (Figure 2). For example, three introgressions on chromosome 1, including IL1-1, IL1-1-2, and L1-1-3, were defined by the RFLP marker CT233 at one end. But in this study, IL1-1 and IL1-1-2 was terminated at SSR1-0.4, and IL1-1-3 was terminated at SSR1-0.6. The physical distance between SSR1-0.4 and SSR1-0.6 was 258.2 kb. In addition, eight introgressions from LA0716 were shorter than the bins framed by the RFLPs, three were much longer and another three were not detected in this study.

We found that the segments between the frames as defined by the SSRs did not match those previously defined by RFLPs. We also found that IL2-3, IL2-4 and IL9-3 were heterozygous in some individuals of LA4038, LA4039, and LA4084, respectively. The heterozygosity of introgressions should lead to shortened introgressions in the selfing progenies. In fact, we observed eight introgressions with reduced sizes defined by SSRs, compared to those defined by RFLPs. Gur *et al*. [14] reported that IL8-1 could lead to lethality if homozygous. In our study, IL8-1 was as long as its derivative introgression IL8-1-1, losing the lethality-induced loci related segment on the chromosome. Besides IL8-1, loci related to sterility also existed in introgressions (such as IL1-1, IL1-2, IL2-1), leading to partial sterility (http://tgrc.ucdavis.edu). This indicated that sterility-related loci on chromosomes should, if not induced, contribute to the formation and persistence of those introgressions. The presence of heterozygous introgressions suggested that some ILs were not yet completely stable.

In this study, 179 SSRs were selected to investigate previously undefined segments in ILs, and a total of 54 previously undefined segments were found in 21 ILs (28% ILs, 21/75) (Figure 2 and Table S2). In the process of developing the ILs, four generation backcrossing and a few generations of self-pollination were carried out to purify the genetic background. This was carried out with the aid of RFLP selection for unique introgressions [1], [6]. We know that it is difficult to remove the all unspecific chromosome segments derived from the wild parent in developed progenies. Therefore, the discovery of undefined segments was expected, and if we applied more consecutive SSRs on a chromosome, more previously undefined segments should be found. In fact, these undefined segments were also detected in the bin maps of the DArT and RFLP markers [11]. However, one undefined segment (75A) was too large (>46837 kb) to be explained by the remaining segment, even after four backcrosses, or by a deficiency in high-density molecular markers during IL development. The probable origin of 75A might be from IL12-2 by an accidental cross between LA4099 and LA4102.

The relative location of each detected introgression in this work agreed with that defined by the RFLPs, but the genetic size of each introgression did not always reflect its physical size, even though we excluded heterochromatin from the analysis. A total of 10 introgressions (13.9%) were observed with RPS/RGS values almost equal to one, 25 introgressions (34.7%) with values of more than one and the remaining 37 (51.4%) had values of less than one (Table S3). In theory, the lower the RPS/RGS value of an introgression, the smaller the introgression should be, due to the elevated recombination frequency. In fact, of 19 introgressions of less than 2000 kb (excluding heterochromatin), 14 (73.4%) introgressions had RPS/RGS values of less than one, two (10.5%) were equal to one, and three (15.8%) were greater than one. Comparatively, among 23 introgressions of more than 5000 kb (excluding heterochromatin), 10 (43.5%) introgressions had RPS/RGS values less than one, four (17.4%) introgressions which were equal to one, and nine (39.1%) introgressions of less than one. Additionally, of 54 previously undefined segments, 26 (48.1%) were located in regions with a RPS/RGS of less than one, and 20 segments (37.0%) located in introgression regions with a RPS/RGS greater than one. Therefore, for these ILs to be used for fine mapping of populations, larger population sizes are needed for those introgressions with high RPS/RGS values to obtain a good segregation of the progenies, rather than introgressions with small RPS/RGS values.

In summary, we report the development of 829 polymorphic SSRs between M82 and LA0716 which we integrated into the physical distance map with 96 RFLPs. These markers were used to define introgressions in the 75 ILs developed by Eshed and Zamir [6]. We found 72 introgressions from LA0716 defined previously by RFLPs; however, three introgressions, including IL2-1, IL6-1 and IL10-3, were not found. Moreover, 54 previously undefined segments with RFLPs were found in 21 ILs, ranging from 1–11 in one IL. The new SSRs, along with the physical distance and IL maps, will facilitate genetic analysis of tomato and future breeding experiments.

## Supporting Information

Table S1 **Characteristics of the 829 mapped SSR markers developed from the released scaffolds of the tomato genome.**
(XLS)

Table S2 **Characteristics of specific introgressions and unspecific segments in 75 introgression lines from the cross between LA0716 (** ***S. pennellii*** **) × cv. M82 (** ***S. lycopersicum*** **).**
(XLS)

Table S3 **Comparative analysis of introgressions in 75 introgression lines (ILs) from the cross between LA0716 (** ***S. pennellii*** **) × cv. M82 (** ***S. lycopersicum*** **).**
(XLS)
